# Expression of human uncoupling protein-3 in *Drosophila* insulin-producing cells increases insulin-like peptide (DILP) levels and shortens lifespan

**DOI:** 10.1016/j.exger.2009.02.001

**Published:** 2009-05

**Authors:** Dickon M. Humphrey, Janne M. Toivonen, Maria Giannakou, Linda Partridge, Martin D. Brand

**Affiliations:** a MRC Dunn Human Nutrition Unit, Wellcome Trust/MRC Building, Hills Road, Cambridge CB2 0XY, UK; b Department of Biology, University College London, Darwin Building, Gower Street, London WC1E 6BT, UK; c Buck Institute for Age Research, 8001 Redwood Blvd., Novato, CA 94945, USA

**Keywords:** *Drosophila*, UCP3, Uncoupling protein, Insulin signalling, Ageing, Lifespan

## Abstract

Uncoupling proteins (UCPs) can dissipate mitochondrial protonmotive force by increasing the proton conductance of the inner membrane and through this effect could decrease ROS production, ameliorate oxidative stress and extend lifespan. We investigated whether ubiquitous, pan-neuronal or neurosecretory cell-specific expression of human UCP3 (hUCP3) in adult *Drosophila melanogaster* affected lifespan. Low, ubiquitous expression of hUCP3 at levels found in rodent skeletal muscle mitochondria did not affect proton conductance in mitochondria isolated from whole flies, but high pan-neuronal expression of hUCP3 increased the proton conductance of mitochondria isolated from fly heads. Expression of hUCP3 at moderate levels in adult neurons led to a marginal lifespan-extension in males. However, high expression of hUCP3 in neuronal tissue shortened lifespan. The life-shortening effect was replicated when hUCP3 was expressed specifically in median neurosecretory cells (mNSC), which express three of the *Drosophila* insulin-like peptides (DILPs). Expression of hUCP3 in the mNSC did not alter expression of *dilp2*, *dilp3* or *dilp5* mRNA, but led to increased amounts of DILP2 in fly heads. These data suggest that lowering mitochondrial coupling by high expression of hUCP3 alters mNSC function in a way that appears to increase DILP-levels in fly heads and lead to a concomitant decrease in lifespan.

## 1 Introduction

The oxidative damage theory of ageing postulates that accumulated damage to macromolecules caused by reactive oxygen species (ROS) ultimately leads to tissue failure and death (Beckman and Ames, 1998). Most ROS produced in animal cells originate from the mitochondria, as a by-product of respiration. Electrons can escape from the respiratory chain and reduce molecular oxygen to form superoxide (Boveris and Chance, 1973; Boveris, 1977). Correlative evidence has come, for instance, from the finding that ROS-production from isolated mitochondria is associated with lifespan across five species of flies (Sohal et al., 1995; Sohal and Weindruch, 1996). Attenuation of ROS production could therefore be one way to ameliorate ageing-related damage. However, other studies have produced contrary findings (Guo et al., 2001; Orr et al., 2003; Bayne et al., 2005). Precise modulations of ROS-production may be required to ameliorate oxidative damage in a way that extends lifespan because ROS are also involved in signalling pathways (D’Autreaux and Toledano, 2007) and immune function (Bogdan et al., 2000). ROS-production by isolated mitochondria is highly sensitive to mitochondrial protonmotive force in both mammals (Liu, 1997; Korshunov et al., 1997; Lambert and Brand, 2004), and *Drosophila* (Miwa et al., 2003). Decreasing protonmotive force by mild uncoupling of mitochondria could attenuate oxidative damage and decrease the rate of ageing (Skulachev, 1996; Brand et al., 2004).

The uncoupling proteins (UCPs) are a family of mitochondrial proteins whose best-characterised function (in the presence of suitable activators) is to cause partial uncoupling of oxidative phosphorylation by dissipating the protonmotive force generated by the electron transport chain. The capacity of UCPs to circumvent endogenous oxidative stress was demonstrated in mitochondria from *Ucp3* knockout mice, which have significantly higher levels of oxidative damage than wild-type controls (Vidal-Puig et al., 2000; Brand et al., 2002). The presence of UCP3 was also found to protect the mitochondrial tricarboxylic acid cycle enzyme aconitase against inactivation by oxidative damage in vitro (Talbot and Brand, 2005). An increase in macrophage ROS production in *Ucp2*^−/−^ mice (Arsenijevic et al., 2000) and the finding that pancreatic islets from *Ucp2*^−/−^ mice show significantly raised mitochondrial membrane potential and superoxide production (Krauss et al., 2003) provide further evidence for in vivo ability of uncoupling proteins to lower levels of mitochondrial ROS. Furthermore, a recent study of over-expression of UCP1 in mouse skeletal muscle showed increased median (but not maximal) lifespan and alteration of several ageing-related pathologies (Gates et al., 2007).

Mitochondrial oxidative stress in neuronal tissue has been implicated in age-associated neurodegenerative diseases, such as Parkinson’s and Alzheimer’s diseases (Jenner, 1993; Finkel and Holbrook, 2000; Wang et al., 2005). Based on observations in *Ucp2*^−/−^ mice, it has been suggested that UCP2 has a role in helping to maintain normal dopaminergic neuronal function (Andrews et al., 2006). UCP2 over-expression significantly decreases infarct size after ischemia reperfusion brain injury in mice (Mattiasson et al., 2003), although, as with most UCP overexpression studies, it is unclear whether this represents a native or an artifactual activity of the UCP2 (Brand and Esteves, 2005). Over-expression of human Cu, Zn-superoxide dismutase in motor neurons of *Drosophila* has been reported to extend lifespan up to 40% in wild-type flies, and also to rescue lifespan by up to 80% in *Sod*^−/−^ flies (Parkes et al., 1998), suggesting that neuronal superoxide production can limit lifespan (but see Ford et al., 2007). More specifically, it was found that inducible, adult-specific expression of hUCP2 in neuronal tissue of *Drosophila* can lead to extension of lifespan, supporting the hypothesis that attenuating production of ROS by mild uncoupling catalysed by UCPs can increase lifespan (Fridell et al., 2005). However, recent studies using cultured mammalian neurons suggest that mild uncoupling is not protective against ROS-production (Johnson-Cadwell et al., 2007; Tretter and Adam-Vizi, 2007).

In mammals, UCP2 also plays a central role as a negative modulator of glucose-stimulated insulin-secretion in pancreatic β-cells (Zhang et al., 2001). Deficiency of UCP2 increases coupling efficiency in clonal β-cells (Affourtit and Brand, 2008) and protects against hyperglycemic (Krauss et al., 2003) and lipid-induced β-cell dysfunction (Joseph et al., 2004). Insulin/insulin-like growth factor (IGF)-like signalling (IIS), which is conserved in the nematode worm, *Caenorhabditis elegans*, the fruit fly, *Drosophila*, and the mouse has recently gained attention for its involvement in the determination of lifespan (reviews: Gems and Partridge, 2001; Garofalo, 2002; Tatar et al., 2003; Giannakou and Partridge, 2007; Piper et al., 2008). Three of the *Drosophila* insulin-like peptides (DILPs) are secreted from the median neurosecretory cells (mNSCs) in the brain, and play a fundamental role in the IIS pathway in *Drosophila* (Ikeya et al., 2002; Rulifson et al., 2002; Giannakou and Partridge, 2007). Partial ablation of this small group of cells leads to a decreased expression of *dilp2*, *dilp3* and *dilp5* mRNA and up to 33% extension of lifespan (Broughton et al., 2005). The *Drosophila* genome does not contain orthologues of UCP2 or UCP3. Recently, however, a *Drosophila* homologue of the brain mitochondrial carrier protein 1 (dBMCP1) was characterised in the heterologous yeast system and was found to exhibit some properties associated with mitochondrial uncoupling (Fridell et al., 2005). There is still some debate as to whether the primary function of BMCP1 and the related UCP4 is mitochondrial uncoupling; especially since BMCP1 and UCP4 are more divergent and are located on the phylogenic tree at a greater distance from other uncoupling proteins, closer to oxoglutarate carriers. Problems commonly associated with overexpression of membrane spanning proteins giving false-positive mitochondrial uncoupling (Brand and Esteves, 2005) led Sánchez-Blanco et al. (2006) to examine the function of dBMCP1 in the fly. Upon knocking out dBMCP1 it was found that the mitochondria displayed the same respiration kinetics to control fly mitochondria and dBMCP1 appeared to play a more major role in metabolic homeostasis and control of metabolism during starvation stress (Sánchez-Blanco et al., 2006). Thus, the importance of mitochondrial mild uncoupling and ROS in insulin-secreting tissues of invertebrates awaits investigation.

In the present work, building on the work of Fridell et al. (2005) using human UCP2, we focused on the expression of human UCP3 (hUCP3) in *Drosophila*, which has not been studied previously in this model, and which has been shown to have a strong phenotype when overexpressed in mammals (Clapham et al., 2000). We used high ectopic expression of hUCP3 as a tool that is known to artificially uncouple mitochondria (Harper et al., 2002; Brand and Esteves, 2005) in order to investigate whether mild uncoupling caused by expression of human UCP3 in *Drosophila* (ubiquitously, pan-neuronally or specifically in the mNSCs) affects lifespan or DILP expression. We find that high, ubiquitous expression of hUCP3 does not increase lifespan, while adult-specific neuronal expression causes a marginal lifespan-extension only in males. In contrast, increasing the amount of neuronal hUCP3 expression to an extent that produces measurable increases in proton conductance results in increased DILP protein levels in head samples and dramatic shortening of lifespan, suggesting that mitochondrial coupling in the mNSCs affects DILP secretion.

## 2 Materials and methods

### 2.1 Fly maintenance and lifespan experiments

*Drosophila melanogaster* were reared and lifespan experiments were conducted on SY food: 50 g sucrose (Tate & Lyle Sugars, London, UK), 100 g brewer’s yeast (MP Biomedicals, London, UK), 15 g agar (Sigma), 3 g Nipagin^®^ M (methyl 4-hydroxybenzoate, Clariant UK Ltd., Pontypridd, UK) and 3 ml propionic acid (Sigma) per litre. Nipagin^®^ M was added in a solution containing 100 g/L Nipagin^®^ M in 95% ethanol. Ubiquitous drivers *actin-GAL4* (stock # 4414) and *da-GAL4* (stock # 8641) were obtained from Bloomington *Drosophila* Stock Center (Indiana, USA). *dilp2-GAL4* was obtained from Dr. Tomoatsu Ikeya (Ikeya et al., 2002; Broughton et al., 2005) and elav-GS was kind gift from Dr. Ronald Davis (Osterwalder et al., 2001). All transgenic lines (including *UAS-hUCP3* lines, below) were backcrossed to existing *w*^1118^ stock for 10–12 generations and tested negative for *Wolbachia* endosymbiont using PCR-based detection, as described in Toivonen et al. (2007). To generate experimental flies, age-matched parents were mated in 2–4 cages containing grape juice agar plates with live yeast paste. After 2 days of acclimatization, eggs laid during a 6 h window were collected, and the same volume of embryos (approximately 300 flies) was transferred to each rearing bottle. Newly eclosed flies were transferred to new bottles without anaesthesia and left for 48 h to mate. Sexes were separated by brief CO_2_ exposure and the flies were transferred into experimental vials (with and without RU486), 10 flies per vial. Fresh food vials were provided three times per week and deaths were counted. Raw data from the lifespan studies is presented in Supplementary Table 2.

### 2.2 Generation of UAS-hUCP3 transgenic flies

*hUCP3* cDNA was amplified and cloned in p{UAST} vector under the control of a yeast *GAL4* upstream activation sequence (*UAS*, Phelps and Brand, 1998). Four independent UAS-hUCP3 lines (2E-yw, 2E-or, 2H and 2A) were obtained by microinjection into *w*^1118^ embryos using standard methods (Spradling, 1986). To produce the *UAS-hUCP3-high* strain, females from line 2A were crossed with males from line 2H (both in chromosome 3). Transheterozygous (*2A/2H*) female progeny were crossed with existing balancer stock *w*^1118^; *CyO/Sp*; *TM6B/MKRS* and resultant *w*^1118^; *+/CyO*; *2A,2H/TM6B* progeny were collected based on intense eye colour indicating the presence of recombinant (*2A,2H*) chromosome. These females were then crossed with *w^1118^; 2E-yw; +/MKRS* males and *w*^1118^; *2E-yw/CyO*; *2A,2H/MKRS* progeny were collected. The stock was finally made homozygous for all three transgenic insertions (*w*^1118^; *2e-yw*; *2A,2H*) and named *UAS-hUCP3-high*.

### 2.3 Preparation of mitochondria

The preparation method for *Drosophila* mitochondria was modified and developed from existing protocols (Miwa et al., 2003). Isolation medium was 250 mM sucrose, 5 mM Tris–HCl, 2 mM EGTA, 1% w/v bovine serum albumin (BSA), pH 7.4 at 4 °C. For isolation of mitochondria from fly bodies, about 300 flies were immobilised by chilling on ice, decanted into a chilled mortar previously rinsed with isolation media and processed in two steps. Firstly, about 2 mL of ice-cold isolation medium was added and the flies were pressed very gently with a pestle to break the soft abdomen and release digestive enzymes. The liquid was pipetted off, and about 0.5 mL of fresh medium was added. Secondly, the flies were pressed more firmly, applying little force and avoiding shearing. The liquid was passed through two layers of absorbent muslin (Robert Bailey & Son plc, Stockport, England) and immediately centrifuged at 150*g* for 3 min in a Sorvall SS-34 rotor at 4 °C. The supernatant was passed through one layer of muslin and re-centrifuged at 9000*g* for 10 min. The supernatant was discarded and the pellet was carefully resuspended in around 60 μL isolation medium (200 μL/g flies) to give about 30 mg protein/mL and kept on ice.

For head mitochondria, flies were anaesthetised with CO_2_ and heads were separated from bodies using a razor blade. Heads were collected in a chilled mortar previously rinsed with isolation medium. The heads were immediately pressed more firmly and then passed through two layers of muslin as before with additional washings of the mortar with 2–3 mL isolation medium. Mitochondria were pelleted as above.

For mouse skeletal muscle mitochondria, isolation medium was 0.1 M KCl, 0.05 M Tris–HCl, 2 mM EGTA adjusted to pH 7.4 at 4 °C. Mitochondria were isolated from the hind leg muscles of wildtype or UCP3 knockout mice essentially as described previously (Cadenas et al., 2002). Approximately 10 g of tissue was used in each isolation, taken from 2 to 3 mice between 8 and 10 weeks of age. Tissue was chopped finely and washed in several changes of isolation medium at 4 °C. The tissue was then partially digested for 3 min in isolation medium supplemented with 1 mM ATP, 5 mM MgCl_2_·6H_2_O, 0.5% w/v fatted BSA and 180.6 units protease (adjusted to pH 7.4 at 4 °C). Partially digested tissue was then homogenised using a Dounce homogeniser and centrifuged at 490*g* in Sorvall SS-34 rotor to remove cell debris and fat. The supernatant was transferred to fresh tubes and mitochondria were collected at 10,368 g and washed twice with isolation medium – once at 10,368 g and again at 3841 g. Mitochondrial pellets were suspended in 50 μL isolation medium. Protein concentration was determined by the Bio-Rad (Richmond, CA, USA) modified Bradford protein assay, with BSA as the standard.

### 2.4 Immunodetection and quantification of hUCP3

**Fig. 1:**
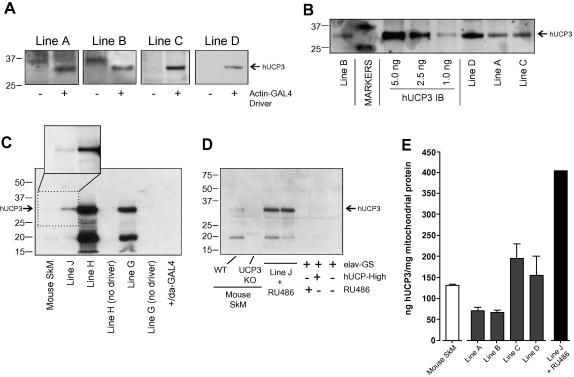
Western detection of hUCP3 protein in fly mitochondria. (A) hUCP3 is expressed in whole body mitochondria from four independent *transgenic* lines (A–D) when driven by actin-GAL4, as detected by immunostaining with anti-UCP3 antibody. The positions of the 25 kD and 37 kD molecular weight standards are indicated. Forty micrograms of mitochondrial protein was loaded in each lane. (B) Western blot used for quantification of hUCP3 levels in whole body mitochondria (20 μg/lane). (C) Expression of hUCP3 at high and low levels in head or whole body mitochondria (40 μg/lane) from different transgenic lines. Lane 1: mouse skeletal muscle mitochondria; lane 2: fly head mitochondria from line J (+RU486); lanes 3–6: fly whole body mitochondria from lines G and H with and without da-GAL4; lane 7: da-GAL4 control. The inset (lanes 1 and 2) shows a longer exposure to highlight UCP3 expression in mouse skeletal muscle. (D) Inducible expression of hUCP3 in head mitochondria (line J) compared to UCP3 expression in wild-type and *Ucp3^−/−^* mouse skeletal muscle mitochondria. Forty micrograms of mitochondrial protein was loaded in each lane. (E) Amount of hUCP3 per mg mitochondrial protein from two (lines A, B, and D) or four (lines C and J) independent mitochondrial samples. Amounts in line J were measured only approximately, assuming linear densitometry responses in panels (C) and (D) and a value of 130 ± 4 ng UCP3/mg mitochondrial protein for mouse skeletal muscle mitochondria (Cadenas et al., 2002; Harper et al., 2002). This literature value for mouse skeletal muscle mitochondria (SkM) is also shown for comparison. Bars show mean values ± SEM or range.

Proteins were separated using SDS polyacrylamide gel electrophoresis (SDS–PAGE). Samples were added at 4 mg protein/mL to loading buffer containing 50 mM Tris–HCl (pH 6.8), 1% w/v SDS, 10% v/v glycerol, 50 mM β-mercaptoethanol and 0.01% w/v bromophenol blue and heated to 90 °C for 5 min. 20 or 40 μg protein per well (as stated in figure legends) was added to 12% acrylamide bis-acrylamide gels. After separation for 1 h at 150 V, protein was transferred to nitrocellulose membranes (Whatman Schleicher & Schuell) using a semi-dry technique (25 V for 30 min). Nitrocellulose membranes were blocked for 30 min with 5% milk powder in Tris–HCl buffered saline containing 0.1% Tween-20 (TBST). Blocked membranes were then washed five times for 5 min in TBST. Primary antibody was applied at 1:2000 anti-UCP3 (ABR) in 5% milk in TBST for 2 h at room temperature. Secondary antibody was used at 1:4000 HRP-linked goat anti-rabbit IGF (Sigma) for 2 h at room temperature. Membranes were then washed in TBST and incubated for 5 min with ECL-plus (GE Healthcare) to visualise protein. Emitted light was detected using Kodak film. The anti-UCP3 antibody also detected a higher molecular-weight band above UCP3 (Fig. 1A). The higher molecular-weight band was present in both *act-GAL4/UAS-hUCP3* flies and *UAS-hUCP3/CyO* controls (line A) and was not detected in additional experiments (Fig. 1B–D). Therefore the band was assumed to be non-specific binding of the anti-UCP3 antibody. A low molecular weight protein was detected in UCP3-expressing flies (Fig. 1C and D) and mouse skeletal muscle mitochondria (Fig. 1D). This band has previously been observed as a cleavage product of UCP3 as a result of freeze-thawing the mitochondrial sample (Harper et al., 2002). To check equal loading on the gels and equal transfer, protein loading was visualised on the membranes using Gelcode (Pierce) according to the manufacturer’s instructions (data not shown). Densitometry of protein loading and hUCP3 was performed using ImageJ (U.S. National Institutes of Health, Bethesda, Maryland, USA). hUCP3 expressed in *Escherichia coli* inclusion bodies was used to calibrate the UCP3 content of fly mitochondria. Inclusion body protein was prepared as described previously (Harper et al., 2002). The final inclusion body pellet was solubilised in 5 mM Mops, 30 mM Na_2_SO_4_ and 1.5% sarkosyl, pH 7.3, for 45–60 min at 20–22 °C. Insoluble material was removed by centrifugation at 27,200*g* for 15 min and solubilised inclusion bodies were stored in aliquots at −85 °C.

### 2.5 DILP-2 immunodetection in Drosophila head samples

Live flies were snap-frozen in liquid nitrogen and stored at −80 °C. Frozen flies of each genotype were shaken for 30–60 s in an Eppendorf microfuge tube. The dismembered flies were tipped onto two stacked stainless steel sieves (Endcotts Ltd., London, England) pre-chilled to −20 °C. The upper sieve was 24 mesh (710 μm opening) and retained the abdomen-thorax; the lower one was 35 mesh (425 μm opening) and retained only the heads. The sieves were shaken by hand for approximately 1 min. Thirty heads per strain were homogenised in 180 μl of 50 mM Tris–HCl (pH 7.5), 150 mM NaCl, 1% w/v SDS, 10% v/v glycerol and 0.5 mM EGTA. Homogenised head samples were centrifuged to collect exoskeletal debris and 100 μL of supernatant was diluted to 2 mg protein/mL in loading buffer with β-mercaptoethanol without boiling. Thirty micrograms of protein was loaded per lane onto 15% acrylamide bis-acrylamide gels. Bio-Rad modified Bradford protein assay was used to estimate protein. Protein was separated and transferred (semi-dry) as above to nitrocellulose, blocked and blotted with 1:5000 rabbit polyclonal anti-DILP2 antibody (kind gift from Priyanka Belawat; Broughton et al., 2005) at room temperature for 2 h. Secondary antibody was 1:8000 HRP-linked goat anti-rabbit as above for 1 h at room temperature. Protein was visualised as before.

### 2.6 Proton leak in fly mitochondria

Proton leak kinetics were measured as the dependence of the rate of oxygen consumption driving proton leak on membrane potential in the presence of 1 μg/mL oligomycin (an inhibitor of ATP synthase, preventing any changes in the respiration rate needed to drive phosphorylation). Standard KHEM incubation medium comprised 120 mM KCl, 3 mM Hepes, 1 mM EGTA and 1 mM MgCl_2_, adjusted to pH 7.2 with KOH at 25 or 30 °C. Mitochondria from whole flies were incubated at 0.35 mg protein/mL in 2 mL KHEM supplemented with 0.3% w/v BSA and 150 μM palmitate, pH 7.2 at 25 °C in a 4 mL Clark-type oxygen electrode (Rank Brothers, Cambridge, England) to measure respiration rates. Mitochondria from fly heads were incubated at 0.6 mg protein/mL in 2 mL KHEM supplemented with 5 mM phosphate, at 30 °C, pH 7.2. Electrode buffer was assumed to contain 479 or 444 nmol O/mL at 25 or 30 °C respectively. The electrode chamber was closed, avoiding air bubbles, and subsequent additions were made by micro-syringe through a small channel. Membrane potential was measured simultaneously using an electrode sensitive to the potential-dependent probe triphenylmethyl phosphonium cation (TPMP^+^) (Brand and Kesseler, 1995). 5 μM rotenone (a complex I inhibitor, to prevent oxidation of endogenous NAD-linked substrates) and 80 ng/mL nigericin (to collapse the pH gradient, allowing the entire protonmotive force to be measured as a membrane potential) were added. The TPMP^+^ electrode was calibrated before each titration with 5 sequential additions of 0.5 μM TPMP^+^. Following calibration, respiration was initiated by adding 20 mM α-glycerol 3-phosphate (neutralised with HCl). After 1.5 min, membrane potential was varied in steps by sequential additions of cyanide (50 mM KCN in 0.5 M Hepes, pH 7.2; final concentrations 5–255 μM). At the end of the titration, 0.6 μM FCCP was added to release TPMP^+^ from the mitochondria to allow baseline correction. Membrane potentials were calculated as described previously, assuming a TPMP^+^-binding coefficient of 0.24 and a matrix volume of 0.67 μL mg^−1^ (Brand et al., 2005).

### 2.7 RNA extraction and real-time quantitative PCR

Total RNA was extracted from heads of 25 frozen flies in 1 ml Trizol (Gibco). A FastPrep FP120 high-speed reciprocating device (Thermo Electron Corporation) was used to disrupt the tissues for 20 s at maximum speed. RNA was separated into the aqueous phase by adding 200 μL chloroform and shaking vigorously for 15 s. The samples were incubated for 3 min at 20 °C then centrifuged at 12,000*g* for 15 min at 4 °C. The aqueous phases were removed to new tubes, mixed with 500 μL isopropyl alcohol and 50 μL NaOAc and incubated at −80 °C for 40 min. Samples were centrifuged at 12,000*g* for 15 min at 4 °C and the supernatants were discarded. Pellets were washed in 700 μL ice-cold 70% v/v ethanol in water containing 0.1% (v/v) diethylpyrocarbonate (DEPC H_2_O). Samples were spun at 9000 g, washed once more with 70% v/v ethanol in DEPC H_2_O and left to air dry for 1 min. Dried RNA pellets were resuspended in 20 μL DEPC H_2_O and stored at −80 °C. Four independent RNA extractions of from heads were performed in triplicate for each genotype: UAS-hUCP3-high/dilp2-GAL4, UAS-hUCP3-high/+ and dilp2-GAL4/+. cDNA was synthesised using Superscript 2 Reverse Transcriptase (Invitrogen) following the manufacturer’s instructions. RNA was incubated for 5 min at 65 °C with thymine oligonucleotide and deoxy-nucleotide triphosphate mix; followed by 2 min at 42 °C; finally RNA samples were incubated for 50 min with Superscript 2 Reverse Transcriptase. The reaction was stopped by heating to 70 °C for 15 min, and then chilled on ice.

Quantitative RT-PCR was carried out as described previously (Broughton et al., 2005). *dilp* transcript levels were normalised to *actin5C*. Primers used were *dilp*2F (TCTGCAGTGAAAAGCTCAACGA) and *dilp*2R (TCGGCACCGGGCATG) for *dilp2*, *dilp*3F (AGAGAACTTTGGACCCCGTGAA) and *dilp*3R (TGAACCGAACTATCACTCAACAGTCT) for *dilp3*, *dilp*5F (GAGGCACCTTGGGCCTATTC) and *dilp*5R (CATGTGGTGAGATTCGGAGCTA) for *dilp5*, and *actin5C*-forward (CACACCAAATCTTACAAAATGTGTGA) and *actin5C*-reverse (AATCCGGCCTTGCACATG) for *actin5C*. The cycle number at which SYBR Green fluorescence exceeds a threshold level was used as a measure of mRNA content. Because of possible variations in efficiency and amplification of reverse transcription, this method provides only an estimate of mRNA copy number, but it allows precise determination of the relative contributions of each *dilp* to the total pool of *dilp* transcripts. A standard curve with 0.125–2 μg cDNA from *dilp2-GAL4/+* fly heads was used to standardise the relative concentrations of *UAS-hUCP3-high/dilp2-GAL4* and *UAS-hUCP3-high/+* fly extracts. Fold-differences were calculated between *UAS-hUCP3-high/dilp2-GAL4* and *UAS-hUCP3-high/+* fly extracts, and *dilp2-GAL4/+* fly extracts.

### 2.8 Statistical analyses

Proton leak calculations were performed in Microsoft Excel. When comparing proton-leak curves at a fixed membrane potential, the FORECAST function was used to calculate respiration rates between data points (vertical dotted line in Fig. 3A). Mean and standard errors in Fig. 3B were calculated from individual repeats. Student’s *t*-test was used to determine significance of differences between two groups of data. When more than two groups of data were compared, variance was measured using one-way ANOVA followed by Tukey’s multiple comparison post tests. The significance confidence limit was set at 95%. Statistical analysis was performed using GraphPad Prism version 4.0a for Mac OS X (GraphPad Software, San Diego California USA). Log-rank tests of survivorship curves were performed using JMP^™^ IN statistical software (SAS Institute Inc.).

## 3 Results

### 3.1 hUCP3 expression in different transgenic lines

To express hUCP3 in *Drosophila*, hUCP3 cDNA was cloned under the control of a yeast *GAL4* upstream activation sequence (*UAS*, Phelps and Brand, 1998). Four independent *UAS-hUCP3* transgenic insertions (named 2E-yw, 2E-or, 2H and 2A) were obtained by microinjection into fly (*w*^1118^) embryos. We first examined hUCP3 expression levels of these transgenes when combined with the ubiquitous driver *actin-GAL4* (lines A–D; Table 1). All crosses produced viable progeny. Mitochondria were isolated from whole *act-GAL4/UAS-hUCP3* flies and from *UAS-hUCP3/CyO* controls. hUCP3 protein was detectable only in the *act-GAL4/UAS-hUCP3* flies (Fig. 1A) and in isolated mitochondria, but not in whole fly homogenates, suggesting hUCP3 is concentrated in the mitochondrial fraction (data not shown).

There was variation in hUCP3 expression levels among the *UAS-hUCP3* lines. To quantify the expression levels, standards purified from inclusion bodies of hUCP3-transformed bacteria (Harper et al., 2002) were included and densitometry was performed on the Western blots (Fig. 1B and E). Over the range studied, the intensity of the standard bands increased linearly as protein concentration was increased (*R*^2^ = 0.95). In the low-expressing lines A and B, hUCP3 content was 71 ± 10 (*n* = 2) and 67 ± 6 (*n* = 2) ng UCP3/mg total mitochondrial protein, respectively. This is approximately half the level of endogenous UCP3 in mouse and rat skeletal muscle mitochondria, which is about 120–140 ng UCP3/mg total mitochondrial protein (Cadenas et al., 2002; Harper et al., 2002). Lines C and D had moderately higher expression, with UCP3 levels of 155 ± 47 (*n* = 2) and 194 ± 37 (*n* = 4) ng UCP3/mg total mitochondrial protein, respectively, comparable to those in mouse and rat.

To create a transgenic line that expressed hUCP3 to a higher level, transgenic insertions in lines 2A and 2H (both in chromosome 3) were joined by recombination and the recombinant chromosome was further combined with insertion 2E-yw in chromosome 2 (see Section 2 for details). The resultant line was made homozygous for the three *UAS-hUCP3* insertions and was named *UAS-hUCP3-high*. Previously, neuronal hUCP2 expression was found to be embryonic lethal in flies (Fridell et al., 2005). When the transgenes from the *UAS-hUCP3-high* were expressed ubiquitously using *act-GAL4* (line I) the offspring also showed increased embryonic lethality (91% and 81% lethality in females (*n* = 204) and males (*n* = 183), respectively). We used this as an indication of increased expression of hUCP3 in the *hUCP3-high/act-GAL4* flies. The offspring of crosses with another ubiquitous driver, *da-GAL4* (line H) were viable. Western blot analysis (Fig. 1C) of *hUCP3-high/da-GAL4 flies* confirmed that the expression of hUCP3 was more than twice as high as in the highest-expressing single insertion transgenic line (line G) using the same driver. Expression was considerably higher than UCP3 in mouse skeletal muscle, which was below the detection limit in the blot in Fig. 1C, but could be resolved in a longer exposure (Fig. 1C inset). The low molecular weight protein detected in both mouse and fly samples (Fig. 1C and D) is a cleavage product that results from freeze-thawing of the mitochondrial sample (Harper et al., 2002). Neuronal expression of hUCP3 during development also increased lethality. Therefore, expression of high levels of hUCP3 was induced post-eclosion in the adult nervous system, using the *elav-G*eneswitch (*elav-GS*) driver (Osterwalder et al., 2001), which expresses modified yeast GAL4 transcription factor that can be selectively activated in neurons by feeding the flies the synthetic progesterone RU486 (Roman et al., 2001). Western blots indicated a 3-fold higher expression of hUCP3 in head mitochondria from this line when treated with RU486 (line J + RU486) compared to endogenous levels in mouse skeletal muscle (Fig. 1D and E). No expression was detected when the inducer RU486 was not present (Fig. 1D).

In summary (Table 1), by using different transgenic *UAS-hUCP3* insertions and their multi-insertion derivative (*UAS-hUCP3-high*), we generated transgenic lines expressing hUCP3 ubiquitously at half (A and B), equal (C and D) or higher (G and H) levels than endogenous levels in mouse skeletal muscle. In addition, we generated lines in which expression of low/moderate levels (E and F) or high levels (J) of hUCP3 were induced pan-neuronally in adults using the *elav-GS* driver. When induced with RU486, high-expresser line J expressed hUCP3 in neurons at about three times the endogenous level in mouse skeletal muscle.

### 3.2 No effect of ubiquitous hUCP3 expression on proton leak in mitochondria isolated from whole flies

**Fig. 2:**
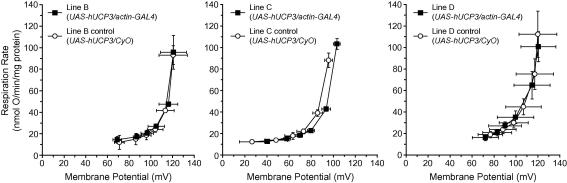
Kinetics of proton leak in whole body mitochondria from ubiquitous hUCP3 expressers (lines B–D). The dependence of respiration driving proton leak on membrane potential was determined as described in Section 2. Data are from two (line B) or three (lines C and D) separate mitochondrial isolations performed in duplicate. Error bars indicate range (line B) or SEM (lines C and D).

We assessed the activity of the hUCP3 protein by measuring proton leak kinetics in mitochondria isolated from whole flies (lines B, C and D) expressing hUCP3 ubiquitously with *actin-GAL4* (Fig. 2). There was no significant change in mitochondrial proton leak kinetics in *actin-GAL4/UAS-hUCP3* lines compared to *UAS-hUCP3/CyO* controls. Moreover, proton conductance was not induced in mitochondria from hUCP3-expressing strains by adding 4-hydroxynonenal, an activator of UCP3 proton conductance activity (Echtay et al., 2003) (data not shown). Maximum membrane potentials were lower than previously reported for *Drosophila* mitochondria (Brand et al., 2005) probably because 150 μM palmitate, previously found to be necessary for UCP3 activation (Echtay et al., 2002), was added to the incubations shown here. Importantly, changes in mitochondrial coupling can still be detected around 120 mV, discounting any effect caused by low membrane potentials (Brand et al., 2005). There was a small increase in state 4 respiration rate in mitochondria from line C compared to the control, which was not due to an increase in proton conductance (if anything, there was a decrease) and was therefore the result of increased respiratory capacity. This conclusion was supported by the observation that mitochondria from line C contained more flavin mononucleotide (FMN), an indicator of respiratory Complex I content, than did control mitochondria (data not shown).

### 3.3 Proton leak in head mitochondria of flies expressing hUCP3 in neurons

**Fig. 3:**
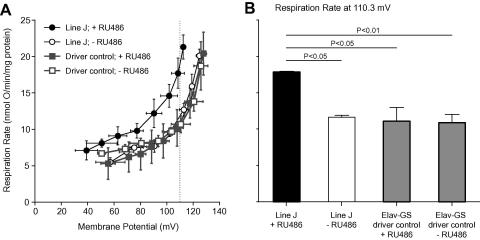
Kinetics of proton leak in head mitochondria from a high neuronal hUCP3 expresser. (A) The dependence of proton leak on membrane potential was determined as described in Section 2. Mitochondria were isolated from heads from UAS-hUCP3-high/elav-GS flies (line J) and +/elav-GS driver control flies with (+) and without (-) RU486. The vertical dotted line indicates the highest potential common to all individual data points (110.3 mV). (B) Respiration rate driving proton leak at 110.3 mV for the four groups. Significance was determined using one-way ANOVA. Data from three separate mitochondrial preparations performed singly or in duplicate.

We next focused on effects of expression of hUCP3 in neurons, because it has been reported that expression of hUCP2 in *Drosophila* neuronal tissue leads to increased proton conductance of head mitochondria and to an extension of lifespan (Fridell et al., 2005). In contrast to whole body mitochondria from flies with low or moderate ubiquitous hUCP3 expression (lines B–D), we found that mitochondria derived from heads of flies expressing high levels of hUCP3 in neurons (line J + RU486) had significantly higher proton conductance than controls not expressing hUCP3 (Fig. 3A). The respiration rate driving proton leak at the highest common potential for all the groups was significantly higher in hUCP3 expressers compared with all controls (mean respiration at 110.3 mV different: *P* = 0.0057; Fig. 3B). An uncoupling activity associated with hUCP3 expression without the requirement for added activators suggests that the protein was misfolded, as reported for skeletal muscle mitochondria from mice overexpressing hUCP3 (Cadenas et al., 2002), resulting in a genuine but non-native increase in proton conductance. Having demonstrated that mitochondrial proton leak was increased in mitochondria from flies with high neuronal expression of hUCP3, we then analysed the effect of hUCP3 expression on lifespan. Because the level of mitochondrial uncoupling could be important for the effects on lifespan, we studied both moderate and high level hUCP3 expressers.

### 3.4 Neuronal and mNSC-specific expression of hUCP3

Superoxide production by mitochondria isolated from mammals (Liu, 1997; Korshunov et al., 1997) or *Drosophila* (Miwa et al., 2003) can be acutely sensitive to membrane potential, so if mitochondrial superoxide production contributes to ageing, mild mitochondrial uncoupling might extend lifespan. Indeed, it has been reported that expression of hUCP2 in fly neurons can lead to increased paraquat resistance and lifespan extension (Fridell et al., 2005). UCP2 and UCP3 are probably functionally similar proteins, judging from proton leak kinetics (Echtay et al., 2003) and studies using proteoliposomes containing UCP2 and UCP3 (Echtay et al., 2001). So, if these in vitro studies apply to the situation in vivo, we might expect similar effects on lifespan in flies expressing either protein.

**Supplementary Fig. 1:**
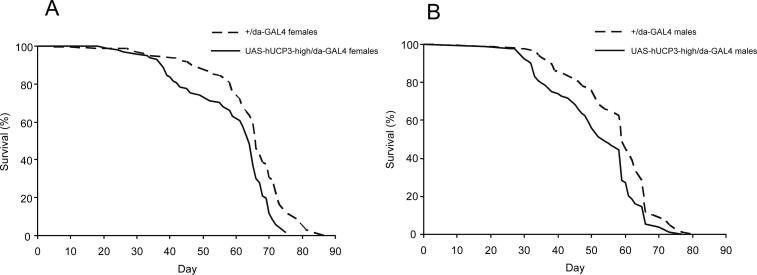


**Fig. 4:**
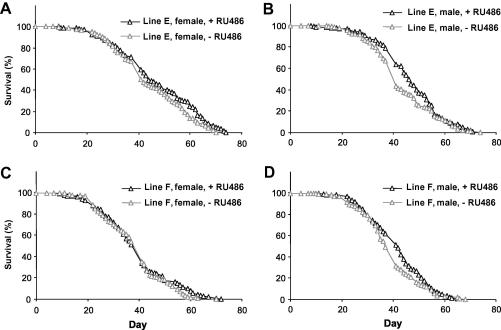
Survival of flies expressing moderate levels of hUCP3 in neurons in the adult. For each panel (A–D) genetically identical UAS-hUCP3/elav-GS flies are shown in induced (+RU486) and non-induced (−RU486) conditions. (A) Line E (UAS-hUCP-2H/elav-GS) females. Median lifespans 44 d (+RU486, *n* = 152) and 42 d (−RU486, *n* = 172). Log-rank test *λ*^2^ = 0.91, *P* = 0.341, median test for maximum lifespan (last surviving 10%), *λ*^2^ = 0.90, *P* = 0.343. (B) Line E (UAS-hUCP-2H/elav-GS) males. Median lifespans 46 d (+RU486, *n* = 156) and 41 d (−RU486, *n* = 164). Log-rank test *λ*^2^ = 3.99, *P* = 0.046, median test for maximum lifespan (last surviving 10%), *λ*^2^ = 0.10, *P* = 0.747. (C) Line F (UAS-hUCP-2A/elav-GS) females. Median lifespans 41 d (+RU486, *n* = 163) and 41 d (−RU486, *n* = 176). Log-rank test *λ*^2^ = 0.99, *P* = 0.319, median test for maximum lifespan (last surviving 10%), *λ*^2^ = 17.58, *P* < 0.0001. (D) Line F (UAS-hUCP-2A/elav-GS) males. Median lifespans 42 d (+RU486, *n* = 161) and 37 d (−RU486, *n* = 159). Log-rank test *λ*^2^ = 3.77, *P* = 0.052, median test for maximum lifespan (last surviving 10%), *λ*^2^ = 0.88, *P* = 0.348.

**Fig. 5:**
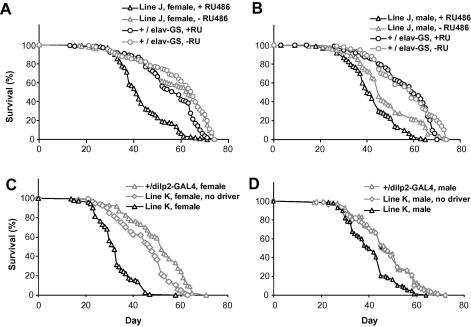
Survival of flies expressing high levels of hUCP3 in neurons or mNSCs in the adult. (A) Pan-neuronal expression of hUCP3-high in line J (UAS-hUCP-high/elav-GS) females. Median lifespans for line J females are 41 d (+RU486, *n* = 128) and 63 d (−RU486, *n* = 117), log-rank test *λ*^2^ = 91.77, *P* < 0.0001. The driver control (+/elav-GS) females show small negative effect on lifespan in the presence of RU486 (log-rank test *λ*^2^ = 18.58, *P* < 0.0001). However, line J females were significantly short-lived also when compared with driver control on +RU486 (log-rank test *λ*^2^ = 65.95, *P* < 0.0001). (B) Pan-neuronal expression of hUCP3-high in line J (UAS-hUCP-high/elav-GS) males. Median lifespans for line J males are 41 d (+RU486, *n* = 96) and 45 d (−RU486, *n* = 95), log-rank test *λ*^2^ = 17.62, *P* < 0.0001. There is no effect of RU486 on lifespan for the driver control males (log-rank test *λ*^2^ = 2.14, *P* = 0.1431). Line J males also show significant effect on lifespan in the absence of RU486. However, under induced conditions line J males are significantly short lived compared with all controls. (C) mNSC-specific expression of hUCP3-high in line K females (UAS-hUCP3-high/dilp2-GAL4, median lifespan 32 d, *n* = 100), driver controls (+/dilp2-GAL4, median lifespan 52 d, *n* = 92, log-rank test *λ*^2^ = 120.11, *P* < 0.0001) and line K without the driver (UAS-hUCP3-high/+, median lifespan 47 d, *n* = 92, log-rank test *λ*^2^ = 73.17, *P* < 0.0001). The two control lines were significantly different in females (log-rank test *λ*^2^ = 19.25, *P* < 0.0001) but both lived significantly longer than line K. (D) mNSC-specific expression of hUCP3-high in line K males (UAS-hUCP3-high/dilp2-GAL4, median lifespan 40 d, *n* = 88), driver controls (+/dilp2-GAL4, median lifespan 46 d, *n* = 83, log-rank test *λ*^2^ = 21.92, *P* < 0.0001) and line K without the driver (UAS-hUCP3-high/+, median lifespan 47 d, *n* = 88, log-rank test *λ*^2^ = 19.64, *P* < 0.0001). The two controls lines were not significantly different in males (log-rank test *λ*^2^ = 0.082, *P* < 0.772).

Pleiotropic effects caused by ubiquitous expression of hUCP3 may be detrimental to lifespan, and may mask any positive, tissue-dependent effects of mitochondrial uncoupling. Indeed, we found no evidence for lifespan-extension when the *da-GAL4* driver was used to induce high ubiquitous hUCP3 expression (in fact the effect was negative, Supplementary Fig. 1). To restrict hUCP3 expression to adult neurons, we used an inducible driver, *elav*-*GS*. No significant effect on female median lifespan was observed in moderate hUCP3 over-expressers (+RU486) when compared with non-induced (−RU486) flies of the same genotype (Fig. 4A and C, for lines E and F, respectively). However, Line F showed small but significant effect on maximum lifespan, as measured from the last 10% surviving flies (Supplementary Table 1). In males, one of the lines (E) showed a small, marginally significant, increase in median lifespan (Fig. 4B), replicated with line F (Fig. 4D), but no effect on maximum lifespan (Supplementary Table 1). As reported earlier in similar experiments (Fridell et al., 2005), the level of uncoupling may be important for lifespan effects. We therefore repeated the experiment using the high hUCP3 expressers. However, stronger expression of hUCP3 in neuronal tissue (line J + RU486) resulted in a dramatic decrease in median and maximum lifespan compared to genetically identical, non-induced controls, the effect being stronger in females than in males (Fig. 5A and B, median lifespans for females 63 d (control) vs. 41 d (induced) and for males 45 d (control) vs. 41 d (induced)). The *elav-GS* driver alone had a small negative effect on female, but not male lifespan on +RU486. However, both line J females and males were significantly short-lived in induced conditions when compared with any controls. Maximum lifespan was also significantly shorter in both sexes in flies where a high level of hUCP3 was induced (Supplementary Table 1).

We investigated further neuronal targets to identify cell types that could be responsible for the decrease in lifespan. The mNSCs are neurosecretory cells, genetic ablation of which decreases the levels of mNSC-expressed *dilp*s and leads to a robust lifespan-extension, presumably by suppression of the insulin-signalling pathway (Broughton et al., 2005). Targeted expression of hUCP3 in mNSCs (Line K), by using the *dilp2-GAL4* driver (Ikeya et al., 2002), resulted in a dramatic decrease in lifespan, similar to that seen with pan-neuronal expression (Fig. 5C and D; median lifespans for females 52 d (driver control) vs. 32 d (over-expresser), and for males 46 d (driver control) vs. 40 d (over-expresser)). The maximum lifespan also was shorter in both sexes in flies that expressed hUCP3 in the mNSCs, compared with both control lines (Supplementary Table 1). The phenotypic similarity of *elav-GS* and *dilp2-GAL4* driven hUCP3-high expressers suggests that the shortened lifespan of pan-neuronal hUCP3 expressers may be accounted for by effects in mNSCs.

### 3.5 mNSC–specific hUCP3 expression does not change dilp transcription but increases DILP2 protein levels

**Fig. 6:**
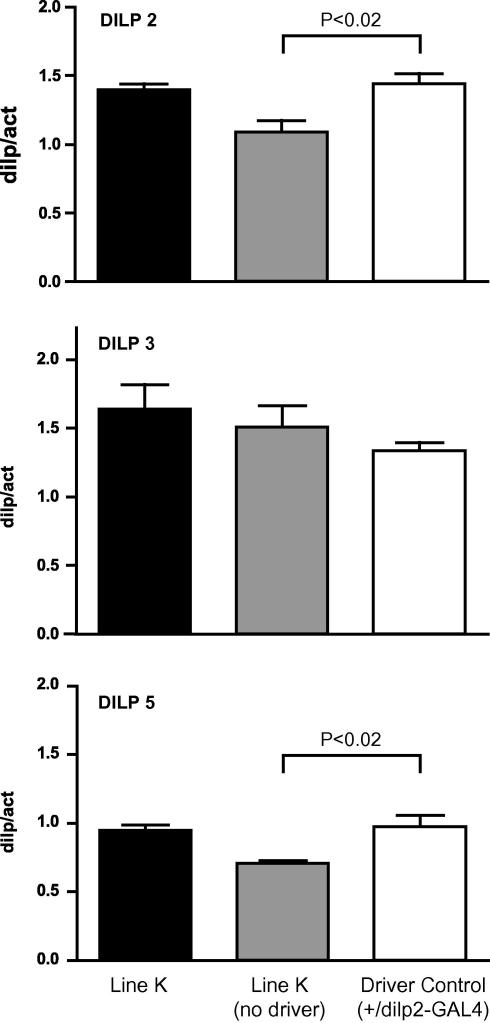
Quantitative PCR analysis of *dilp2*, *dilp3* and *dilp5* mRNA from fly heads. Transcripts were assayed from flies expressing hUCP3 in mNSCs and from controls. Levels are expressed relative to actin5C mRNA levels. Black bars: line K (UAS-hUCP3-high/dilp2-GAL4), grey bars: line K without the driver (UAS-hUCP3-high/+), white bars: driver control (+/dilp2-GAL4). Data are from four independent fly head preparations, each performed in triplicate. Significance was determined by one-way ANOVA.

If hUCP3 expression caused the median neurosecretory cells to fail, the expression of *dilp* mRNAs should be reduced and lifespan would be expected to increase (Broughton et al., 2005). However, expression of hUCP3 in the mNSCs had the opposite effect on lifespan. We used quantitative real-time PCR on fly head samples to measure *dilp2*, *dilp3* and *dilp5* mRNA levels in hUCP3-expressing and control strains. There was no significant difference between hUCP3 expresser line K compared with *+/dilp2-GAL4* driver control, although the driver caused a small but significant change in *dilp2* and *dilp5* (but not *dilp3*) gene expression (Fig. 6; *dilp2*, *P* = 0.013; *dilp5*, *P* = 0.016 by one way ANOVA). However, post-hoc analysis showed that there was no significant driver effect on DILP2 protein levels (see below). If we take *dilp* gene expression level as a marker of mNSC viability, this was not significantly affected by the expression of hUCP3.

**Fig. 7:**
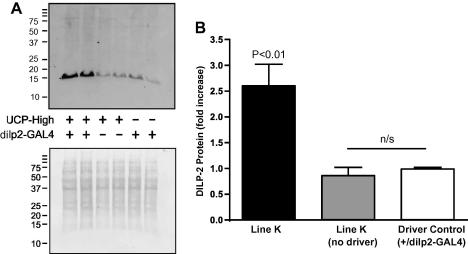
Expression of DILP2 peptide in flies expressing hUCP3 in mNSCs. (A) DILP2 protein visualised by anti-DILP2 antibody (upper panel); membrane from the same blot stained with Coomassie blue (lower panel). Expression of DILP2 was compared to the same control genotypes as in Fig. 6. (B) Quantification of DILP2 protein level relative to driver controls, measured by densitometry. Intensity was corrected for protein loading using the intensity of the same blot stained with Coomassie blue. 30 heads per genotype were used per sample. Thirty micrograms of protein was loaded per lane. Bars show mean ± SEM from three replicates. Western blots each loaded with two independent samples of 30 heads per genotype. Significance was determined by one-way ANOVA.

Since DILPs are secreted hormones, and protein levels may not correlate with gene expression, we investigated whether expression of hUCP3 altered the levels of DILP peptides present in fly heads. DILP2 protein in UAS-*UCP3-high/dilp2-GAL4* flies (line K) and *UCP3-high/+* and *+/dilp2-GAL4* controls was quantified by immunostaining. DILP2 is a 137 amino-acid peptide (GenBank accession number AAF50204), molecular weight 15.26 kD (calculated using the Sequence Manipulation Suite, Stothard, 2000). Western blots probed with anti-DILP2 antibody (Broughton et al., 2005), revealed a single band around 15 kD, suggesting that the antibody specifically detected DILP2 (Fig. 7A, representative sample from three replicate immunoblots). Densitometric quantification (Fig. 7B) showed that mNSC-targeted expression of hUCP3 resulted in a large (2.6 ± 0.4-fold) increase in DILP2 levels compared to control strains. Importantly, the *dilp2-GAL4* driver did not significantly affect the level of DILP2 compared to the *UCP3-high/+* strain.

## 4 Discussion

Mild decreases in mitochondrial protonmotive force caused by uncoupling proteins have been postulated to attenuate endogenous oxidative stress and protect against ageing (Skulachev, 1996; Brand, 2000). Fridell et al. (2005) showed that expression of hUCP2 specifically in *Drosophila* neurons significantly extends lifespan. Thus, mild uncoupling of mitochondria in fly neurons may indeed lower ROS-production and protect flies against ageing. The same authors, however, found the effect to be tissue specific and noted that ubiquitous expression of hUCP2 failed to extend lifespan, failing to support the postulate (Fridell et al., 2005). These findings are supported by data from *Caenorhabditis elegans* where knockdown of *Cel-Ucp4* – present only in head muscles, body wall muscles and pharynx – results in changes in ATP levels and a small increase in mitochondrial membrane potential, but does not result in lifespan shortening (Iser et al., 2005). Similarly, we found previously that mild mitochondrial uncoupling by ubiquitous over-expression of the mitochondrial adenine nucleotide translocase (ANT) in *Drosophila* decreased ROS-production in isolated mitochondria but did not lead to lifespan-extension (Miwa et al., 2004). Furthermore, in the present study, moderate expression of hUCP3 in fly neurons did not extend lifespan in females, while the effect on males was borderline (Fig. 4). High-level ubiquitous expression of hUCP3 failed to extend lifespan (supplementary Fig. 1). However, we did not measure ROS levels in these flies. It may be that ROS levels were not greatly decreased, explaining the small lifespan effect. Indeed, mild uncoupling in cultured mammalian neurons, caused by chemical uncouplers, does not lower the production of ROS, arguing against the postulate (Johnson-Cadwell et al., 2007; Tretter and Adam-Vizi, 2007).

The uncoupling effect of UCP3 reported here differs from the effect of hUCP2 reported by Fridell et al. (2005). Moderate ubiquitous expression of hUCP3 did not uncouple mitochondria (Fig. 2), while Fridell et al. (2005) reported increased respiration following moderate UCP2 expression. However, they did not directly measure proton leak, but a surrogate – respiration rate – that can be affected by electron transport chain content, as can be seen in Fig. 2B (state 4 rates). They used GDP to test for active, folded UCP2, but inhibition by GDP is not specific for UCP2 because it also inhibits uncoupling through ANT (Parker et al., 2008). The third marker that suggests UCP2 is active and uncoupling mitochondria is the effect on lifespan demonstrated by Fridell et al. (2005). Conversely, when we confirmed mitochondrial uncoupling in neuronal mitochondria, we saw lifespan attenuation. At least some of these differences might be explained by differences in expression levels, although it should be noted that in our experiments UCP3 was expressed at the levels found in mouse muscle mitochondria, while Fridell et al. (2005) did not quantify UCP2 expression levels.

True uncoupling of mitochondria, which we demonstrate here in mitochondria from fly heads, may interfere with additional neuronal systems. It is possible secondary deleterious effects resulting from ubiquitous mild uncoupling activity may be overcoming any beneficial tissue-specific effects of decreased ROS production, providing an explanation for the lifespan effects. When hUCP3 expression in fly neurons was increased to about three times the native levels found in rodent muscle, lifespan was dramatically curtailed. Since UCP2 and UCP3 are thought to be functionally similar (Echtay et al., 2001, 2003), the present work suggests that there are secondary factors that affect lifespan due to mild uncoupling, which are independent of ROS.

When high-level mNSC-specific expression was used, short lifespan was accompanied by increased DILP2 protein levels in fly heads, suggesting that production and/or secretion of DILPs from median neurosecretory cells can be modulated by changes in mitochondrial coupling. Up-regulation of IIS caused by a 2.6-fold increase in DILP2 concentration could explain the short lifespan of these flies. However, it is equally possible that other mNSC-expressed DILPs (DILP3 and DILP5, not tested) contribute to or are responsible for the curtailed lifespan. Overexpression of all *Drosophila* DILPs can promote larval growth, indicating that they act as *Drosophila* insulin receptor agonists (Ikeya et al., 2002). Conversely, decreased DILP levels can lead to lifespan extension (Hwangbo et al., 2004; Broughton et al., 2005). It remains to be formally demonstrated which DILPs are restricting normal lifespan and we have not established a biochemical link between the increase in DILP2 protein and an increase in IIS.

Currently, nothing is known about post-transcriptional regulation of DILPs. It remains entirely possible that yet uncharacterised regulatory mechanisms operate translationally or post-translationally (protein degradation, other protein-level modifications, storage, secretion) that contribute to the observed increase in DILP protein levels in the head samples. Furthermore, little is known about the secretory mechanism of DILP-producing cells. The way that mNSCs respond to mitochondrial uncoupling appears to be the converse of the pancreatic β-cell response. UCP2 is present in mammalian pancreatic β-cell mitochondria and its activity lowers mitochondrial oxidative phosphorylation and cytoplasmic ATP/ADP ratios, and blunts insulin secretion in response to glucose (Zhang et al., 2001; Krauss et al., 2003; Affourtit and Brand, 2008). The mNSCs could function in a similar manner to other neurosecretory cells that release hormones (or neurotransmitters) in response to increased cytoplasmic calcium at the pre-synaptic terminal (Burgoyne and Morgan, 1995). Mitochondria can modulate catecholamine release from chromaffin cells by controlling the intracellular levels of Ca^2+^ that trigger exocytosis; uncoupling mitochondria can increase stimulated secretion by three- to five-fold (Montero et al., 2000). It has been proposed that depolarisation of the nerve mitochondria can increase transmitter release by decreasing Ca^2+^ sequestration (David and Barrett, 2003; Talbot et al., 2003). Therefore, partial mitochondrial uncoupling, as a result of high hUCP3 expression could, in theory, lead to increases in cytoplasmic Ca^2+^ and trigger increased DILP secretion. However, we have no direct evidence that the increased DILP2 protein in the head samples results in increased DILP secretion. Since the DILP2 antibody used does not recognize a protein of expected size in the hemolymph samples (Nazif Alic, personal communication), we were not able to study if the peripheral DILP2 levels were affected. Therefore, it remains possible that secretion of the DILPs is actually lowered and the increased protein levels in the heads of the hUCP3-expressing flies reflect accumulation of the DILP2 in the mNSCs. In this case, however, we would expect a positive effect on lifespan, but we observe decreased survival.

Mitochondrial uncoupling in mNSCs probably did not compromise mNSC viability, since DILP gene expression was retained. Furthermore, Broughton and colleagues have shown that disruption of the mNSC increases lifespan (Broughton et al., 2005), while expression of hUCP3 exclusively in the mNSC results in a lifespan shortening. Therefore, this suggests hUCP3 is not causing the mNSC to become ill or to die. ROS production is very sensitive to mild uncoupling in isolated *Drosophila* mitochondria (Miwa et al., 2003) and mild mitochondrial uncoupling may be neuroprotective. Over-expression of UCP2 in cultured cortical neurons caused 50% less caspase 3 activation, and overexpression in vivo increased resistance to neuronal cell death following stroke and brain trauma (Mattiasson et al., 2003). As a result, mild uncoupling may have protected the mNSCs from oxidative stress, improving viability and DILP levels. Alternatively, anti-apoptotic effects of mitochondrial uncoupling in neurons might protect mNSCs or favour their proliferation, and could be responsible for enhanced DILP protein levels in hUCP3 flies.

It is apparent that members of the wider family of UCP homologues, including dBMCP1 (UCP5), have diverse roles which may be relevant to ageing. In addition to attenuation of ROS production, these may include metabolic homeostasis (such as regulating insulin release, UCP2), mitochondrial maintenance or thermogenesis (UCP1) (Wolkow and Iser, 2006). In this study we have highlighted the potential role mitochondrial uncoupling plays in neuron function and the consequences for lifespan. The demonstration that mitochondrial coupling in mNSCs can modulate DILP levels in *Drosophila* has potentially important implications for the study of superoxide production, insulin signalling and ageing. Exogenous hUCP3 expression provides a tool that can be used to modulate and hence promote understanding of the function of the mNSC in organism growth, metabolic homeostasis and lifespan. The mechanism by which mitochondrial uncoupling modulates DILP levels is unknown and confirmation of the potential involvement of reactive oxygen species or Ca^2+^ concentrations are exciting avenues for further exploration.

## Figures and Tables

**Table 1:** Properties of the eleven *Drosophila* lines expressing hUCP3.

Line	UAS-hUCP3 gene	Driver	Site of expression	Mitochondrial UCP3 protein content	Embryonic lethal?	Proton conductance	Lifespan (female)		Lifespan (male)	
							Median	Maximum	Median	Maximum
A	2E-yw	actin-GAL4	Ubiquitous	71 ± 10 ng/mg protein	No	No change	–	–	–	–
B	2E-or	actin-GAL4	Ubiquitous	67 ± 6 ng/mg protein	No	No change	–	–	–	–
C	2H	actin-GAL4	Ubiquitous	194 ± 37 ng/mg protein	No	No change	–	–	–	–
D	2A	actin-GAL4	Ubiquitous	155 ± 47 ng/mg protein	No	No change	–	–	–	–

E	2H	elav-GS	Pan-neuronal	N/D	No	N/D	–	–	+12%	–
F	2A	elav-GS	Pan-neuronal	N/D	No	N/D	–	+9%	–	–

G	2H	da-GAL4	Ubiquitous	High	No	N/D	–	–	–	–
H	High	da-GAL4	Ubiquitous	2x line G	No	N/D	−3%	−13%	−9%	–
I	High	actin-GAL4	Ubiquitous	N/D	Partial	N/D	–	–	–	–

J	High	elav-GS	Pan-neuronal	High	Partial	Increased	−54%	-20%	-10%	−19%
K	High	dilp-2-GAL4	Median neurosecretory cells	N/D	No	N/D	−47%	-33%	-18%	−14%

Lifespan columns indicate change in lifespan compared to control flies not expressing hUCP3. –, indicates no significant change in lifespan. N/D, not determined. For details of the statistical analysis of lifespans see Supplementary Table 1.
