# Supplementary material for: Expression of human uncoupling protein-3 in Drosophila insulin-producing cells increases insulin-like peptide (DILP) levels and shortens lifespan
Source: Exp Gerontol. 2009 May;44(5):316–27. doi: 10.1016/j.exger.2009.02.001 (PMC2698063; doi:10.1016/j.exger.2009.02.001)
Supplement: Supplementary Table 1 [file mmc1.doc]

|  |  |  |  | Log Rank | |  |  |  | Median test | |
| --- | --- | --- | --- | --- | --- | --- | --- | --- | --- | --- |
|  | n= | Median | Change % | χ2= | P= |  | max | Change % | χ2= | P= |
| 2A/elav-GS +RU fem | 163 | 41 | - | 0.99 | 0.319 |  | 63 | +9% | 17.58 | <0.0001 |
| 2A/elav-GS -RU fem | 176 | 41 |  |  |  |  | 58 |  |  |  |
| 2H/elav-GS +RU fem | 152 | 44 | - | 0.91 | 0.341 |  | 66 | - | 0.90 | 0.343 |
| 2H/elav-GS -RU fem | 172 | 42 |  |  |  |  | 67 |  |  |  |
| 2A/elav-GS +RU male | 161 | 42 | - | 3.77 | 0.052 |  | 59 | - | 0.88 | 0.348 |
| 2A/elav-GS -RU male | 159 | 37 |  |  |  |  | 57 |  |  |  |
| 2H/elav-GS +RU male | 156 | 46 | +12% | 3.99 | 0.046 |  | 65 | - | 0.10 | 0.747 |
| 2H/elav-GS -RU male | 164 | 41 |  |  |  |  | 64 |  |  |  |
| hUCP3-hi / elav-GS fem, +RU | 128 | 41 | -54% | 91.77 | <0.0001 |  | 61 | -20% | 17.90 | <0.0001 |
| hUCP3-hi / elav-GS fem, -RU | 117 | 63 |  |  |  |  | 73 |  |  |  |
| hUCP3-hi / elav-GS male, +RU | 96 | 41 | -10% | 17.62 | <0.0001 |  | 59 | -19% | 19.00 | <0.0001 |
| hUCP3-hi / elav-GS male, -RU | 95 | 45 |  |  |  |  | 70 |  |  |  |
| hUCP3-hi / dilp2-3-Gal4 fem | 100 | 32 | -63% | 120.11 | <0.0001 |  | 45 | -42% | 19.00 | <0.0001 |
| + / dilp2-3-Gal4 fem | 92 | 52 |  |  |  |  | 64 |  |  |  |
| hUCP3-hi / dilp2-3-Gal4 fem | 100 | 32 | -47% | 73.17 | <0.0001 |  | 45 | -33% | 19.00 | <0.0001 |
| hUCP3-hi / + fem | 92 | 47 |  |  |  |  | 60 |  |  |  |
| hUCP3-hi / dilp2-3-Gal4 male | 88 | 40 | -15% | 21.92 | <0.0001 |  | 58 | -9% | 12.16 | 0.0005 |
| + / dilp2-3-Gal4 male | 83 | 46 |  |  |  |  | 63 |  |  |  |
| hUCP3-hi / dilp2-3-Gal4 male | 88 | 40 | -18% | 19.64 | <0.0001 |  | 58 | -14% | 19.00 | <0.0001 |
| hUCP3-hi / + male | 88 | 47 |  |  |  |  | 66 |  |  |  |
| hUCP3-hi / da-GAL4 female | 92 | 64 | -3% | 14.26 | 0.0002 |  | 70 | -13% | 10.95 | 0.0009 |
| + / da-GAL4 female | 88 | 66 |  |  |  |  | 75 |  |  |  |
| hUCP3-hi / da-GAL4 male | 86 | 54 | -9% | 8.76 | 0.003 |  | 66 | - | 0.79 | 0.383 |
| + / da-GAL4 male | 81 | 59 |  |  |  |  | 66 |  |  |  |

**Supplementary Table 1** Statistics for the presented lifespan experiments. Differences in death rates at all ages (Log Rank) was assessed by Log Rank test and significance for values of maximum lifespan (max, indicates final surviving 10% for each population) was assessed by the nonparametric median test. Abbreviations used: n, number of flies; median, median day of lifespan; max, 90^th^ percentile of the lifespan; change %, positive (+) or negative(-) change caused by hUCP3 expression compared with the control.
